# Ionic Liquid-Based Optical and Electrochemical Carbon Dioxide Sensors

**DOI:** 10.3390/s151229813

**Published:** 2015-12-04

**Authors:** Kamalakanta Behera, Shubha Pandey, Anu Kadyan, Siddharth Pandey

**Affiliations:** 1 Department of Chemistry, Indian Institute of Technology Delhi, Hauz Khas, New Delhi 110016, India; kamala.iitd@gmail.com (K.B.); anu.kadyan1@gmail.com (A.K.); 2 Department of Science and Technology, Ministry of Science and Technology, Technology Bhawan, New Mehrauli Road, New Delhi 110016, India; shubha.p@nic.in

**Keywords:** ionic liquids, carbon dioxide, sensors, optical sensors, electrochemical sensors

## Abstract

Due to their unusual physicochemical properties (e.g., high thermal stability, low volatility, high intrinsic conductivity, wide electrochemical windows and good solvating ability), ionic liquids have shown immense application potential in many research areas. Applications of ionic liquid in developing various sensors, especially for the sensing of biomolecules, such as nucleic acids, proteins and enzymes, gas sensing and sensing of various important ions, among other chemosensing platforms, are currently being explored by researchers worldwide. The use of ionic liquids for the detection of carbon dioxide (CO_2_) gas is currently a major topic of research due to the associated importance of this gas with daily human life. This review focuses on the application of ionic liquids in optical and electrochemical CO_2_ sensors. The design, mechanism, sensitivity and detection limit of each type of sensor are highlighted in this review.

## 1. Introduction

**Figure 1:**
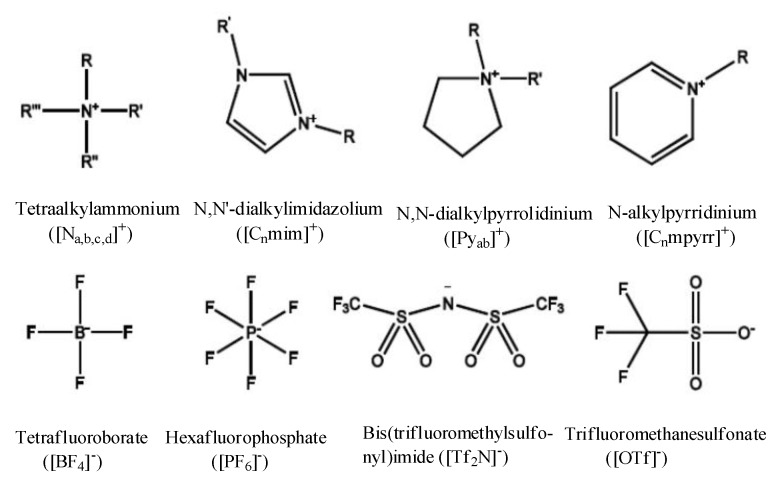
Structures of cations and anions of some commonly-used ionic liquids.

Ionic liquids belong to an exciting class of solvents and have received increased attention from both academic and industrial research communities all over the world as a replacement for conventional solvents in a wide range of applications [1,2,3,4,5]. Ionic liquids are defined as salts with their ions weakly coordinated and remain in the liquid state at temperatures below 100 °C. They are typically comprised of a bulky organic cation (e.g., alkyl-substituted ammonium, imidazolium, pyrrolidinium, *etc.*) paired with an inorganic or organic anion (e.g., halide ions, tetrafluoroborate, hexafluorophosphate, acetate, *etc.*) (Figure 1). Ionic liquids possess several archetypal properties, such as low volatility, high thermal and chemical stability, good electrical conductivity, wide electrochemical windows, wide liquid range, high polarity and good ability to dissolve a wide range of compounds [6,7,8,9,10]. Their negligible vapor pressure and non-flammability have contributed to their common epithet as “green solvents”, and as a result, they have been viewed as alternative replacements for the hazardous and volatile organic solvents largely used in diverse research areas. Ionic liquids are often termed as designer solvents, as their physicochemical properties can be tuned just by tuning the structure of the cation and/or anion [6,7,8,9,10]. Thus, one can synthesize an ionic liquid for a specific application simply by manipulating its key physicochemical properties as a result of the appropriate selection of cation and anion combinations. This key feature, along with their dual behavior as electrolyte and solvent, make them an exciting candidate to be used in diverse research areas of science and technology. These environmentally-benign designer solvents find immense applications in various fields, namely in wide range of synthetic, catalytic and biochemical reactions, separation and extraction processes, as energetic materials, in pharmaceutics, biotechnology, as lubricants, heat transfer fluids, in biocatalysis, nanoscience, among many others [11,12,13,14,15,16,17,18]. Ionic liquids have also become recognized as ideal alternative electrolytes for use in many electrochemical devices, such as batteries, capacitors, fuel cells and solar cells [19,20,21,22]. In addition to the above applications, ionic liquids are vastly used in the sensing of biomolecules (e.g., nucleic acids, proteins and enzymes), various relevant gases (e.g., O_2_, CO_2_, CO, SO_2_, H_2_S, NO_2_, Cl_2_), several ions of importance and sensing of explosives and chemical warfare agents (CWAs) [23,24,25,26,27,28,29,30,31,32].

One of the major problems receiving increased attention is the high levels of carbon dioxide (CO_2_), which have been implicated in climate change and have threatened not only numerous economies, but also the environment of the planet. CO_2_ gas generally produced by fossil fuel combustion and by living beings is a greenhouse gas responsible for global warming. CO_2_ is dangerous to human lives when present at high concentrations. The U.S. Occupational Safety and Health Administration consider a CO_2_ concentration above 4% as “dangerous to life and health”. In addition to environmental monitoring, a variety of other applications exist where knowing the CO_2_ concentration is essential. The continuous and accurate monitoring of CO_2_ levels is of vital importance in environmental monitoring, respiratory analysis, sea water analysis, medicine and biological processes [33,34,35,36]. The development of a sensor with high sensitivity and selectivity that is capable of detecting CO_2_ and quantifying its concentration in a reliable and inexpensive manner is of significant importance to both the medical profession and socially-responsible industries [37,38].

The intrinsic properties of ionic liquids mentioned earlier suggest that these can be potentially used as an advantageous media for the development of stable and robust CO_2_ gas sensors. Ionic liquids possess high thermal stability and low volatility, meaning that they have the ability to sustain high temperatures and pressures, thus enabling them to perform under conditions where conventional organic solvents would struggle to remain physically and chemically unchanged, and as such, can be used where organic solvents would fail [1,2,3,4,5,6,7,8,9,10]. They exhibit wide electrochemical windows, which allow for the investigation of compounds that may have been inaccessible otherwise [39,40,41,42]. Further, their dual behavior as electrolyte and solvent makes them an exciting candidate for sensing purposes [23,24,25,26,27,28,29,30,31,32]. One of the added benefits of using these designer solvents is that they offer many options for chemical modifications, allowing greater flexibility in designing molecular recognition sites in their structures. These possibilities offer opportunities to explore the applications of ionic liquids in the highly sensitive and selective determination of trace amounts of CO_2_ gas using them in sensor arrays [28,29,30]. For example, a task-specific ionic liquid with an amine (–NH_2_) group on the cation can capture CO_2_ and significantly enhance the solubility of CO_2_ in that particular ionic liquid [43]. A wide range of ionic liquid-based sensors has been developed so far for the detection and quantification of CO_2_ gas [44,45,46,47,48,49,50,51,52,53,54,55,56,57,58,59,60,61,62,63,64,65,66,67,68,69,70]. These ionic liquid-based CO_2_ sensors are either optical-based [44,45,46,47,48,49,50,51,52,53,54,55,56,57] or electrochemical-based sensors [58,59,60,61,62,63,64,65,66,67,68,69,70].

Optical CO_2_ sensors based on the changes in the absorbance or fluorescence response have been reported by researchers [44,45,46,47,48,49,50,51,52,53,54,55,56,57]. Usually, these optical sensors contain a chemically-active sensing agent, which changes the UV-VIS absorbance or fluorescence emission intensity in response to CO_2_. Oter *et al.* used ionic liquids 1-methyl-3-butylimidazolium tetrafluoroborate and 1-methyl-3-butylimidazolium bromide in optical CO_2_ sensing together with an ion pair form of acid base indicator, bromothymol blue [44,45,46,47,48]. Water-miscible ionic liquids were exploited as an optical sensor matrix material for sensing of both gaseous and dissolved CO_2_. They revealed photophysical and photochemical properties of the 8-hydroxy-pyrene-1,3,6-trisulfonate (HPTS) in ionic liquids, as well as the performance of the sensor for dissolved and gaseous CO_2_. Further, Ertekin *et al.* reported an optical CO_2_ sensor with ionic liquid-doped electrospun nanofibers based on the changes in the fluorescence signal intensity of an ion pair form of HPTS [49,50]. Further, Wolfbeis *et al.* performed semi-quantitative determination of CO_2_ by dissolving the pH indicators bromothymol blue, thymol blue, and HPTS along with a reference fluorophore in the silicone-ionic liquid emulsions [51]. Tang *et al.* reported a carbamate ionic liquid-based CO_2_ sensor based on the change in the fluorescence response [52]. Further, Pandey *et al.* developed a fluorescent CO_2_ sensor relying on the response of fluorophores 1,3-bis(1-pyrenyl)propane (BPP), 1,3-bis(1-pyrenyl)decane (BPD) and 6-(1-pyrenyl)hexyl-11-(1-pyrenyl)undecanoate (BPHU) [53].

Other than these optical-based sensing methods, electrochemical techniques are shown to have applications for CO_2_ sensing [58,59,60,61,62,63,64,65,66,67,68,69,70]. There are mainly three types of electrochemical sensors based on the measurement of redox current (amperometric), or the development of a potential (potentiometric), or a change in the electrical impedance (conductometric). In the case of amperometric sensors, the analyte species diffuses through the electrolyte to be detected at the working electrode surface [28,58,59]; whereas potentiometric sensors consist of a membrane that contains ion exchangers, lipophilic salts and plasticizers, and the trans-membrane potential affords the information of the activity of the analyte ion in solution. Ionic liquids are a better choice to replace conventional electrolyte systems in amperometric/potentiometric sensors due to their good electrical conductivity and, more importantly, to their wide electrochemical windows [39,40,41,42]. An electrochemical CO_2_ sensor consists of a working electrode with other electrodes (e.g., reference electrode) connected through an electrolyte, which is covered by a gas-permeable membrane. The gas passes through the membrane, diffuses through the electrolyte and is detected at the working electrode. It is shown that the ionic liquid-based electrochemical CO_2_ sensors reported so far are either based on the reaction between oxygen (O_2_) and CO_2_ or on the reduction of CO_2_ gas within ionic liquids, or ionic liquid-polymer-based, or ionic liquid-nanomaterial-based systems. Compton *et al.* reported an electrochemical CO_2_ sensor based on the reduction of CO_2_ within ionic liquid 1-butyl-3-methylimidazolium acetate ([BMIM][Ac]) [60]. They made use of a two-electrode cell (Pt as the working electrode and Ag as the reference electrode) for the chronoamperometric detection of CO_2_ within the ionic liquid. Further, they reported a voltammetric CO_2_ sensor based on the reduction of O_2_ in the presence of CO_2_ within two ionic liquids, 1-ethyl-3-methylimidazolium bis(trifluoromethylsulfonyl) imide ([EMIM][Tf_2_N]) and hexyltriethylammonium bis(trifluoromethylsulfonyl)imide ([N_6222_][Tf_2_N]) [61]. Shimoyama *et al.* reported an electrochemical impedance-based CO_2_ gas sensor using an ionic gel formed by the mixture of an ionic liquid 1-ethyl-3-methyl-imidazolium tetrafluoroborate ([EMIM][BF_4_]) and a polymer polyvinylidene fluoride-cohexafluoro propylene (PVDF-HFP) [62]. Further, they reported ionic liquid-based electrochemical CO_2_ sensors based on carbon nanotubes (CNTs) and graphene, respectively [66,67].

This review focuses on a detailed assessment into the applications of ionic liquids in optical and electrochemical sensors for the sensing of CO_2_ gas.

## 2. Ionic Liquids in Optical CO_2_ Sensors

The use of ionic liquids in optical CO_2_ sensor development is explored by many researchers. In most cases, optical spectroscopic techniques (e.g., UV-VIS absorbance, fluorescence and surface plasmon resonance) have been used as tools for sensing CO_2_. A change in UV-VIS absorbance or reflectance or fluorescence emission intensity within ionic liquid-based systems forms the basis for CO_2_ detection.

Ertekin *et al.* reported a new optical CO_2_ sensor based on the change in the fluorescence signal of 8-hydroxypyrene-1,3,6-trisulfonic acid trisodium salt (HPTS) in ionic liquids [44]. Ionic liquids 1-methyl-3-butylimidazolium tetrafluoroborate ([MBIM][BF_4_]) and 1-methyl-3-butylimidazolium bromide ([MBIM][Br]) were used as matrix materials with HPTS in the above optical CO_2_ sensor. It was observed that the fluorescence intensity of HPTS at 519 and 521 nm decreased with the increasing concentrations of CO_2_ by 90% and 75% in ionic liquids [MBIM][BF_4_] and [MBIM][Br], respectively. The response times of the sensing reagents were observed to be in the range of 1–2 min for switching from N_2_ to CO_2_ and 7–10 min for switching from CO_2_ to N_2_. They also reported another optical CO_2_ sensor based on the spectrophotometric signal changes of another probe, a bromothymol blue/tetraoctylammonium (BTB^−^/TOA^+^) ion pair, in the same above ionic liquids [45]. This sensor was based on the determination of the acidity constant (p*K*_a_) of the modified BTB within the employed ionic liquids. Further, they proposed an emission-based CO_2_ sensor based on the response of an ion pair form of HPTS in ionic liquid 1-ethyl-3-methylimidazolium tetrafluoroborate ([EMIM][BF_4_]) containing ethyl cellulose (EC) matrix [46]. The utilization of ionic liquid [EMIM][BF_4_] in ethyl cellulose matrix is seen to result in superior spectral characteristics, showing an atypical isoemmissive point in modified EC matrix at 418 nm. Next, they developed an optical dissolved CO_2_ sensor based on the spectrofluorimetric signal changes of the fluorescent carbazole within the above [EMIM][BF_4_]^−^ containing ethyl cellulose (EC) matrix [47]. It is important to mention that the above sensor shows a very good detection limit of 500 nM in the form of [HCO_3_]^−^ for dissolved CO_2_.

The development of electrospun nanofiber-based optical CO_2_ sensors was first reported by Ertekin *et al.* [48,49,50]. They used polymethylmethacrylate (PMMA) and ethyl cellulose (EC) as polymeric materials and electrospinning to fabricate optical chemical sensing agents. Electrospinning is found to be a promising, simple and effective method for fabricating optical chemosensor devices. The authors optimized the conditions for electrospinning to form nodal-free PMMA or EC-based continuous nano-fibers by varying the concentrations of plasticizer dioctyl phthalate (DOP), PMMA or EC and ionic liquid 1-ethyl- 3-methylimidazolium tetrafluoroborate ([EMIM][BF_4_]) in the solutions. It was observed that the presence of the ionic liquid in the PMMA solutions facilitates the electrospinning of nodal-free nanofibers due to the high conductivity of the ionic liquid-doped precursor polymer solutions. This electrospun nanofiber-based optical CO_2_ sensor is based on the change in the fluorescence response of an ion pair form of 8-hydroxypyrene-1,3,6-trisulfonic acid (HPTS) in the presence of CO_2_ [48]. This sensor design showed high sensitivities due to the high surface area-to-volume ratio of the nanofibrous membrane structures. The results obtained from Stern–Volmer analysis show that the sensitivities of electrospun nanofibrous membranes to detect CO_2_ are 24–120-fold higher than those of the common thin film-based sensors. More importantly, it was observed that the response times of the sensing reagents were short, and the signal changes were fully reversible. The stability of the ion pair form of HPTS in the employed matrix materials was excellent, and when stored in the ambient air of the laboratory, no significant drift in signal intensity was observed, even after a few months. The presence of ionic liquid [EMIM][BF_4_] is seen to enhance the photostability of HPTS in the polymer matrix. The authors also reported another electrospun nanofiber for the detection of dissolved CO_2_ using the same ionic liquid [EMIM][BF_4_] [49]. In this work, they used a novel fluorescent indicator dye in ethyl cellulose-based plasticized matrix material in the form of electrospun nanofibers. Fluorescent dye *N*′-[(*E*)-(4-nitrophenyl)methylidene]pyridine-4-carbohydrazide was doped together with ionic liquid [EMIM][BF_4_] in the presence of perfluoro acids for fluorescence detection of CO_2_. Further, they proposed a similar electrospun nanofiber-based CO_2_ sensor based on the spectrophotometric signal change of fluorophore bromothymol blue (BTB) [50]. The effect of ionic liquids with different anionic and cationic parts, *i.e.*, [EMIM][BF_4_] and [BMIM][PF_6_], on the sensor signal was tested thoroughly. It is noticed that fluorophore BTB also satisfies the requirement of a distinguishable color change to the naked eye when encapsulated in nanofibers. The above-described nanofiber-based CO_2_ sensor can be used for quantitative determination of CO_2_ in the concentration range of 0.0%–100.0% pCO_2_. With respect to continuous thin films, the electrospun nanofibers are seen to have enhanced sensitivity extending to a 98% relative signal change, lower detection limits and shorter response times.

Wolfbeis *et al.* reported a novel optical CO_2_ sensor based on the emulsion of ionic liquids in a silicone matrix prepared from the mixture of tetravinyltetramethylcyclotetrasiloxane, vinyl-terminated PDMS and methylhydrosiloxane–dimethylsiloxane copolymer [51]. The ionic liquids used are 1-butyl-3-methylimidazolium tetrafluoroborate ([BMIM][BF_4_]) and 1-butyl-3-methylimidazolium tosylate ([BMIM][OTs]). Semiquantitative determination of CO_2_ was achieved from the UV-VIS absorption response of pH indicator thymol blue (TB) and bromothymol blue (BTB) and the fluorescence response of 8-hydroxypyrene-1,3,6-trisulfonate (HPTS) within the ionic liquids. It is noteworthy to mention that the sensitivity of the system can be determined by the p*K*_a_ of the indicator used.

**Figure 2:**
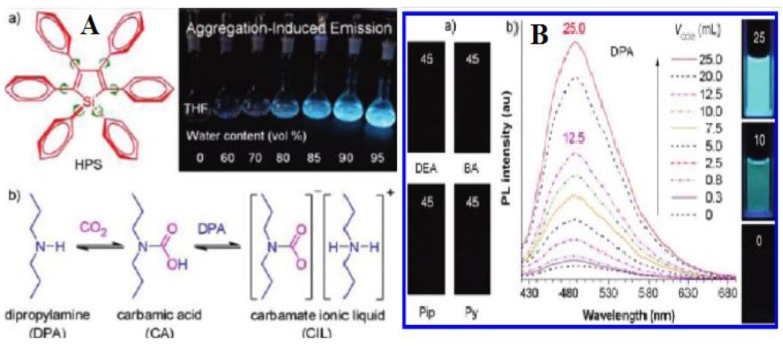
(**A**) (**a**) Hexaphenylsilole (HPS) is nonemissive when it is dissolved in THF, but becomes strongly fluorescent when the molecules are aggregated in the THF/water mixtures with high water contents; (**b**) the formation of carbamate ionic liquid (CIL) by bubbling CO_2_ gas through dipropylamine (DPA); (**B**) (**a**) Photographs of HPS solutions (∼37 μM) in the amines (2 mL) bubbled with CO_2_ gas (45 mL); DEA = diethylamine; BA = butylamine; Pip = piperidine; Py = pyridine; (**b**) PL (Photoluminescence) spectra and photographs of HPS in DPA before and after bubbling with different volumes of CO_2_ (*V*_CO2_). All of the photographs were taken under UV illumination ([52], reprinted (adapted) with permission from Tang B.Z. *et al.* (2010) J. Am. Chem. Soc. 132: 13951–13953. Copyright (2010) American Chemical Society).

A carbamate ionic liquid-based fluorescent chemosensor for the detection and quantitation of carbon dioxide gas was developed by Tang *et al.* [52]. Initially, they noticed that hexaphenylsilole (HPS) is non-luminescent when it is dissolved in THF, but becomes strongly fluorescent when the HPS molecules are aggregated in the THF/water mixtures with high water contents (Figure 2A). They further observed that bubbling CO_2_ through an amine yields a carbamate ionic liquid (CIL), which is accompanied by increases in the polarity and viscosity of the medium. Keeping this in mind, an amine solution of HPS was purged with a stream of CO_2_ gas that turned on the light emission of HPS as its molecules clustered in and its restriction of the intramolecular rotation (RIR) process was activated by the polar and viscous CIL. A series of amines were tested, and it was shown that bubbling large volumes of CO_2_ gas through HPS solutions in piperidine (Pip), pyridine (Py), diethylamine (DEA) and butylamine (BA) caused no recognizable changes in the emission of HPS. However, a green light was emitted from a dipropylamine (DPA) solution of HPS immediately after it had been bubbled with a small volume of CO_2_ gas. Interestingly, the fluorescence intensity of HPS was seen to be increased on increasing the volume of CO_2_ gas. Further, a CIL was prepared by purging DPA with a large excess of CO_2_ gas. A monotonic increase in the fluorescence intensity was observed on increasing the amount of the CIL in DPA solution of HPS (Figure 2B). The plot of the fluorescence intensity *vs.* the concentration of CIL shows a linear trend, which clearly indicates that it is the CIL that has affected the fluorescence emission of HPS. In order to quantify the fraction of CO_2_ in a gas mixture, CO_2_/N_2_ mixtures were used as a model system to check how the fluorescence response of HPS varies with the concentration of CO_2_. The mixtures of CO_2_/N_2_ with varying CO_2_ concentrations were bubbled through HPS solutions in DPA at a fixed rate for a fixed time. It was noticed that the fluorescence intensity of HPS was increased monotonously with increasing concentration of CO_2_. The plot of log intensity *vs.* [CO_2_] gives a straight line over the whole concentration range that enables quantification of CO_2_ under various conditions. It has been reported that bicarbonate is formed in the reaction of amine with CO_2_ in the presence of water [71]. As the conventional CO_2_-sensing processes are susceptible to water, moisturized CO_2_ gas was prepared to examine the effect of water on the performance of sensing. It is noticed that purging a DPA solution of HPS with the moisturized gas shows identical behavior to those obtained by using dry CO_2_ as the bubbling gas. Further, it is observed that the addition of water droplets into DPA solutions of HPS gives almost similar results. Hence, it became clear that the CO_2_-sensing process is not affected significantly by the presence of water. More importantly, the CIL-based sensing scheme is free of the CO-interfering problem, because it is well known that CO does not react with amine [72]. The overall principle of the above CO_2_-sensing mechanism lies in the fact that bubbling DPA liquid with CO_2_ gas results in the formation of a viscous and polar CIL with poor solvating power toward HPS. It is obvious that a viscous medium hinders intramolecular movement, and a polar solvent induces hydrophobic solutes to aggregate. These two effects activate the restriction of the intramolecular rotation process of HPS, thus blocking its nonradiative decay channels and turning on its light emission in the CIL mixture. Increases in CO_2_ concentration increase the viscosity and polarity, thus enabling the gas quantitation. This CO_2_-sensing scheme is observed to permit quantitation of CO_2_ amount over the whole concentration range (0%–100%), and this can be highly appealing, particularly for the areas of seismology and volcanology, where field tracking of the gas mixtures with high amounts of CO_2_ plays a crucial role in disaster prediction and prevention.

Further, Pandey *et al.* reported a fluorescence excimer-based CO_2_ detection strategy using the above carbamate ionic liquid (CIL) system based on the modulation in the local viscosity and polarity within the system [53]. They prepared an optically-responsive switchable CIL by the reaction of two moles of dipropylamine (DPA) with one mole of CO_2_. As it was earlier shown that CO_2_ reacts with DPA to result in conversion into a CIL, the viscosity, as well as polarity of the medium changes significantly, which forms the basis of CO_2_ detection [52]. Taking into account these facts, the authors used excimer-forming fluorescence microviscosity probes 1,3-bis(1-pyrenyl)propane (BPP), 1,3-bis(1-pyrenyl)decane (BPD) and 6-(1-pyrenyl)hexyl-11-(1-pyrenyl)undecanoate (BPHU), respectively, for detection purposes. It is observed that the excimer-to-monomer fluorescence intensity ratio (I_E_/I_M_) decreases linearly with the volume of CO_2_ to which the sample was subjected, thus clearly indicating an increase in the sample viscosity with the increase in CO_2_ volume. Furthermore, the I_E_/I_M_ ratio is observed to decrease linearly with the flow rate of CO_2_. Further, they used a solvatochromic probe, Reichardt’s dye 30, whose absorption band maximum is known to be highly sensitive to both the dipolarity/polarizability and the hydrogen bond donating (HBD) acidity of the milieu. Interestingly, it is noticed that the dye behavior changes dramatically, showing different appearances in neat DPA (pale yellow) *versus* that in CO_2_-saturated DPA (deep purple). This is well supported by the results obtained from the UV-VIS absorbance spectral behavior of the above system.

Based on the above principle of aggregation-induced emission (AIE) that allows molecules to switch from a non-fluorescent to a fluorescent nature, Wang *et al.* reported an amidine-based CO_2_ chemosensor [54]. The chosen amidine-based system became an ionic liquid with high viscosity by bubbling CO_2_ through it as a result of the reaction of amidine with CO_2_. A liquid mixture composed of tetraphenylethene (TPE), 5-amino-1-pentanol (APN) and 1,8-diazabi-cyclo-[5,4,0]-undec-7-ene (DBU) was prepared. TPE and DBU are amidine-containing molecules and show AIE. It was observed that after bubbling with a small volume of CO_2_, the TPE-containing mixture of DBU and APN (1:1, v/v) remained transparent and emitted strong fluorescence immediately. Interestingly, it was noticed that the intensity of fluorescence was increased linearly with the increase in the CO_2_ concentration, thus making it feasible to monitor the amount of CO_2_ quantitatively. Further, the same experiment was carried out with three different volume proportions of DBU to APN, 1:1, 2:1 and 3:1, respectively, to gain insight into the nature of the material. It was observed that the fluorescent intensity of the samples bubbled with CO_2_ for different times (*i.e.*, 0–200 s) increased with increasing the purging time of CO_2_ for all three samples. The sample with a volume ratio of 1:1 was seen to exhibit a linear increase, as well as the strongest fluorescence intensity, as compared to the other two samples with proportions of 2:1 and 3:1, respectively. It is noteworthy that the aforementioned fluorescent chemosensor is observed to have unique sensitivity towards CO_2_, is free of CO interfering problems and has extraordinarily high water-resistant properties.

Yoon *et al.* developed a novel optical CO_2_ sensor that utilized tetrapropyl (TBBI)-based ionic liquids [55]. It was earlier shown that benzobisimidazolium (BBI)-based ionic liquids can act as biscarbene ligands for metal ions, and in some cases, these are found to be fluorescent, as well [73,74,75]. A key feature associated with the BBI-based fluorophores is that functionalization of the C_1_ position of the imidazolium rings can tune their photophysical properties. Further, it was reported that *N*-heterocyclic carbenes (NHCs), which are often obtained by deprotonating the corresponding imidazolium salts, are capable of activating CO_2_ to form imidazolium carboxylates [76,77]. Considering these factors, the authors imagined that the addition of fluoride anion, which acts as a weak base, would produce a species possessing a partial NHC character and, therefore, would allow for the reaction with CO_2_; the changes in the optical features would then enable detection. Keeping this in mind, a specific organic-soluble NHC precursor, *i.e.*, tetrapropyl benzobisimidazolium salt (TBBI), was synthesized, and the TBBI solution in acetonitrile containing three equivalents of F^−^ ions (added as the tetrabutylammonium salt, TBAF) was bubbled with CO_2_ gas. It was observed that on increasing volume of CO_2_ (governed by a mass flow controller), the fluorescence intensity gradually increased, and no additional changes in the emission intensity were observed after the mixture got saturated at a certain CO_2_ concentration. The aforementioned approach using TBBI-based ionic liquids effectively established a new method for optical detection of CO_2_ with fast response times, a low detection limit (*ca*. 30 ppm for this system), very good sensitivity and provides for both fluorescence and colorimetric outputs. Further, they reported a fluorescent and colorimetric CO_2_ sensor using polydiacetylene (PDA)-functionalized imidazolium-based ionic liquid [56]. It was earlier reported that PDAs exhibit a color transition from blue to red that can be triggered when stimuli disrupt the effective polymer conjugation length, typically by inducing a change in the preferred backbone conformation [78]. This phenomenon associated with PDAs is seen to be highly useful in developing various chemosensors [78]. This sensor design relies on the same event, *i.e.*, blue to red color phase change induced by the reaction of CO_2_ with alkylamines tethered to the imidazolium PDA. It was noticed that the reaction of the tethered amine with CO_2_ to form carbamoate salt releases a proton, which is then reacted to another amine molecule, and the generated ammonium ions are unreactive toward CO_2_. The positive charges of the ammonium ions neutralize the newly-formed anionic carbamoate sites, and hence, the aqueous solution of the PDA does not show any color change upon exposure to CO_2_ gas. However, the addition of an external base triethylamine (TEA) to the above system results in the formation of deprotonated ammonium carbamoate that does not interact with the PDA in the absence of CO_2_. This is clearly evident by the lack of change in its visible spectra and the unchanged blue color of the solution. Interestingly, a transform in the color from blue to red is immediately apparent when the above aqueous PDA solution in the presence of TEA is bubbled with a small volume of CO_2_ gas. This is well-supported by the results obtained from UV-VIS and fluorescence spectral changes of the system at different volumes of CO_2_. It is noteworthy that the cationic imidazolium groups play a significant role in the blue to red phase transition in the presence of CO_2_. When aqueous solutions of PDA-NH_2_, having a backbone similar to that of the above PDA and primary amine side chains, but lacking imidazolium moieties, are bubbled with CO_2_ in the presence of TEA, the changes in the spectral behavior are observed to be negligible, and the original blue color remains unchanged. Hence, it is clear that the appended primary amines and the formation of carbamoate anions are not sufficient criteria to induce a PDA color transition. It is important to mention that the above PDA/TEA system is observed to be sufficiently sensitive toward atmospheric CO_2_ detection (*ca*. 400 ppm).

Other than the use of UV-VIS absorption and fluorescence emission-based methods for CO_2_ sensing, a report is found to have used surface plasmon resonance (SPR) for the detection of CO_2_ [57]. Shimoyama *et al.* designed a CO_2_ gas sensor using an SPR-based sensing scheme [79] with ionic liquid [EMIM][BF_4_]. These SPR sensors are generally based on the detection of the permittivity change of the specimen near the sensing surface. The authors fabricated the CO_2_ sensor using an SPR sensing scheme by dropping the ionic liquid [EMIM][BF_4_] on the surface of a prism coated with an Au thin layer. Ionic liquid [EMIM][BF_4_] was used, as it has selective absorbability towards CO_2_ gas. It was observed that the permittivity of the liquid increased when it absorbs CO_2_ gas. This change in permittivity on changing the concentration of CO_2_ was clearly detected by the SPR sensing scheme that forms the basis of CO_2_ detection. Since an SPR dip angle shifts by the change in the permittivity of the specimen, it is noticed that the SPR dip angle of the above sensor shifts when CO_2_ gas is absorbed. It is noteworthy that the above proposed SPR sensor can measure CO_2_ gas with a concentration as low as 700 ppm.

## 3. Ionic Liquids in Electrochemical CO_2_ Sensors

In addition to ionic liquid-based optical CO_2_ sensors, reports also exist on the ionic liquid-based detection of CO_2_ that utilizes electrochemical methods.

Compton *et al.* reported an ionic liquid-based electrochemical CO_2_ sensor that utilizes the reduction of CO_2_ in the ionic liquid [BMIM][acetate] [60]. They used a two-electrode cell with a 10-mm diameter Pt working electrode and a 0.5-mm diameter Ag quasi-reference electrode. Microscale samples of the ionic liquid [BMIM][acetate] containing the cobaltocenium/cobaltocene (Cc^+^/Cc) as the internal reference were held under vacuum conditions to remove water and other gaseous solutes before being exposed to dry CO_2_. It was observed that CO_2_ in [BMIM][acetate] was reduced to the radical anion at −1.3 V *versus* Cc^+^/Cc. It is important to mention that in the acetate ionic liquid, CO_2_ was reduced chemically and irreversibly via one electron transfer followed by the formation of possibly oxalate ion, carbon monoxide and/or carbonate ion. A high solubility (1520 mM) of CO_2_ gas in the ionic liquid was determined from chronoamperometry, whereas the diffusion coefficient measured was an order of magnitude less than that measured in other imidazolium ionic liquids. It is surprising considering the viscosities of the liquids (112, 140 and 371 cP for imidazolium ionic liquids with [BF_4_]^−^, [acetate]^−^ and [PF_6_]^−^ anions, respectively, at 298 K), where in the absence of any follow-up chemical processes, the least viscous ionic liquid is expected to present the fastest diffusion. The reason for enhanced solubility and slowed diffusion is likely due to the chemical complexation of CO_2_ in [BMIM][acetate], thus demonstrating a means for the possible sequestration of CO_2_ gas. Further, authors reported an electrochemical gas sensor for the detection of CO_2_ gas based on the reduction of oxygen (O_2_) in the presence of CO_2_ at a gold microdisk electrode in ionic liquids 1-ethyl-3-methylimidazolium bis(trifluoromethylsulfonyl)imide ([EMIM][Tf_2_N]) and hexyltriethylammonium bis(trifluoromethylsulfonyl)imide ([N_6222_][Tf_2_N]), respectively [61]. From the electrochemical studies, it was shown that on increasing concentration of CO_2_, cyclic voltammetry (CV) shows an increase in the reductive current and reduction of the oxidative current, indicating that the generated superoxide readily reacts with CO_2_. The reaction mechanism between superoxide and carbon dioxide involves the heterogeneous transfer of an electron to dioxygen at the gold working electrode surface. The above observation of the increase in the reductive current on addition of CO_2_ likely results from slower reaction kinetics in the ionic liquid medium as compared to that in conventional aprotic solvents due to the relatively higher viscosity associated with ionic liquids. They have also determined the diffusion coefficient (2.3×10^−10^ m^2^/s) and solubility (55 mM) of CO_2_ within ionic liquid [N_6222_][Tf_2_N] using chronoamperometry. The CO_2_ solubility is observed to be relatively high as compared to more traditional aprotic solvent media, thus suggesting that ionic liquid-based systems may be more advantageous for the detection of such gases.

Ishizu *et al.* have reported an electrochemical CO_2_ gas sensor based on an ionic gel formed by mixing of ionic liquid 1-ethyl-3-methylimidazolium tetrafluoroborate ([EMIM][BF_4_]) and polymer polyvinylidene fluoride-co-hexafluoro propylene (PVDF-HFP) [62]. An organic solvent *N*,*N*-dimethyl acetamide (DMAC) was used to dissolve [EMIM][BF_4_] and PVDF-HFP in the formation of the gel, because PVDF-HFP was insoluble in neat [EMIM][BF_4_]. Finally, DMAC was evaporated by heating the solution to form the ionic liquid gel. The above-prepared [EMIM][BF_4_]-based ionic gel was patterned onto the electrodes. The thickness of the ionic gel was observed to be dozens of times thinner than that of the neat [EMIM][BF_4_] droplet due to the lower viscosity associated with the gel as compared to the neat ionic liquid. Ionic liquid [EMIM][BF_4_] was chosen for the ionic gel-based CO_2_ sensor, as it was observed that [EMIM][BF_4_] selectively absorbs CO_2_ gas. The above electrochemical CO_2_ sensor consisted of two Pt electrodes patterned under the ionic gel. The concentration of CO_2_ gas was detected by measuring the change in the electrochemical impedance between these two electrodes. The observed change in the electrochemical impedance was mainly due to the chemical reaction taking place on electrodes in response to the absorption of CO_2_ gas. Earlier, Rosen *et al.* investigated the chemical reaction taking place as a result of an applied potential to [EMIM][BF_4_] in CO_2_-containing atmosphere [80,81]. They have shown that the increase in the concentration of CO_2_ gas enhances the reduction on the cathode because the cation of the ionic liquid [EMIM] worked as a catalyst in the CO_2_ gas reduction. Further, they have observed that the reduction occurred only when the applied electrical potential to [EMIM][BF_4_] was within a certain range. The above ionic gel-based electrochemical CO_2_ sensor relies on the same sensing principle reported by Rosen *et al.* The change in the electrical impedance is observed to be dependent on the concentration of CO_2_ gas and the applied voltage. To examine the reaction between the ionic gel and CO_2_ gas on the electrode, the authors conducted experiment using a potentiostat/galvanostat by applying a DC voltage to the cell in the range of 0–3.0 V and measuring the current flowing through the cell. They have shown that the current change took place in the range 0.5–2.5 V and that the observed change was caused by the CO_2_ gas reduction that occurred near the electrodes. More importantly, it is observed that this reaction is anticipated to lead to the change of the cell’s impedance, as well. The observed impedance change rate is seen to be proportionally decreasing with the increase in the concentration of CO_2_ gas. Finally, the authors examined the sensitivity of the above ionic gel-based CO_2_ sensor and observed that the above impedance-based electrochemical sensor can measure the difference of a 500-ppm CO_2_ gas concentration by using a power as small as 0.65 μW. Further, Shimoyama *et al.* proposed a low power consumption electrochemical CO_2_ gas sensor using some imidazolium-based ionic liquids [63]. The electrical impedance of the ionic liquids was observed to decrease with the increase in the concentration of CO_2_ gas. Thus, the authors have proposed that the CO_2_ gas concentration of the atmosphere can be easily estimated by measuring the impedance of the ionic liquids. This sensor device has a power consumption of several tens of microwatts only and is lower than the conventional sensor by 1/1000. The resolution of the above proposed sensor was found to be 100 ppm with a short detection time. This electrochemical CO_2_ senor has low power consumption and, hence, can be applied for green energy management.

Zeng *et al.* designed an *in situ* electrochemical quartz crystal microbalance (EQCM)-based CO_2_ sensor, which utilized electrochemical reactions between O_2_ and CO_2_ in three structurally-different ionic liquids: 1-butyl-3-methylimidazolium bis(trifluoromethanesulfonyl)imide ([BMIM][Tf_2_N]), 1-butyl-2,3-dimethylimidazolium bis(trifluoromethanesulfonyl)imide ([BdMIM][Tf_2_N]) and 1-butyl-1methylpyrrolidinium bis(trifluoromethanesulfonyl)imide ([BMPY][Tf_2_N]) [64]. They observed that the QCM integrated with the electrochemical method is significantly more sensitive and powerful for the characterization of the subtle differences (mass change or viscoelastic change) on the ionic liquid/electrode interface as compared to the single electrochemical method. In the formation of the sensor, ionic liquids were drop-coated onto the working electrode made up of Au quartz crystal, and platinum wire and silver wire were used as the counter electrode and quasi-reference electrode, respectively. All potentials were measured using Fc/Fc^+^ (50 μM ferrocene (Fc) in ionic liquids) as the internal reference. From the cyclic voltammetric (CV) response, it was noticed that in the absence of O_2_, CO_2_ was reduced via a chemically-irreversible one electron transfer to CO_2_^•−^ radical anion at around −2.3 V *vs.* Fc/Fc^+^. More importantly, the cathodic peak current was seen to increase linearly with the increase in CO_2_ concentration. In the presence of O_2_, the cathodic peak current for the first cycle at −1.2 V increases, whereas the corresponding anodic peak for superoxide re-oxidation decreases, thus suggesting that the generated superoxide rapidly reacts with CO_2_. It was observed that the typical voltammetric response for CO_2_ at −2.3 V remained absent. From the above CV results, it is clear that the reaction of CO_2_ and superoxide has taken place rapidly, and there is a competition between ionic liquid cations and CO_2_ to react with the electrochemically-generated superoxide. It was noticed that CO_2_ reduction in ionic liquids is irreversible and forms CO_2_^•−^ adsorbate at the electrode interface. On increasing the concentrations of CO_2_, the reduction of O_2_ is switched from a one-electron process to an overall two-electron process and forms adsorbed CO_2_^•−^ intermediate species. Even though the mechanisms of the electrochemical reaction between CO_2_ and electrochemically-generated superoxide radical (O_2_^•−^) are found to be similar in all three ionic liquids, simultaneously performed EQCM experimental results show that the structurally different cation-based ionic liquids can modify the kinetics of the electrode reactions of O_2_ and CO_2_ due to a competition between the ionic liquid cation and CO_2_ to react with O_2_^•−^. It was observed that the reactivity of O_2_^•−^ toward CO_2_ follows the order of the stability of the ionic liquid cation under the O_2_^•−^ attack as [BMPY][Tf_2_N] > [BdMIM][Tf_2_N] > [BMIM][Tf_2_N]. This EQCM technique-based sensor is found to be a very good tool for CO_2_ detection in terms of the electrochemical reduction of CO_2_ in ionic liquids.

Further, Masel *et al.* reported an ionic liquid-based microfabricated CO_2_ sensor that operates at room temperature with very low power input [65]. The device uses the selective adsorption of CO_2_ in ionic liquid 1-ethyl-3-methylimidazolium tetrafluoroborate ([EMIM][BF_4_]) followed by an electrochemical reaction at a gold electrode to detect the presence of CO_2_. The sensor substrate was made from a 100-mm silicon wafer on which a 500-nm thermal oxide layer was grown. On the silicon wafer, 17-nm chromium and 100-nm gold layers were deposited as electrodes. A small droplet of the ionic liquid was painted between the source and drain electrodes. The [EMIM][BF_4_] droplet was painted over the entire lateral surface of the junction to protect the electroactive portion of the sensor. The potential between the source and the drain electrode was scanned from 0–5.5 V at a scan rate of 100 mV/s. The sensing mechanism for this ionic liquid-based microfabricated sensor is based on the reduction of CO_2_ at the cathode and the subsequent oxidation of that species at the anode. It was observed that the device can measure normal atmospheric levels of CO_2_ with high reliability using less than 10 μW of power. It is important to mention that the low selectivity of [EMIM][BF_4_] to O_2_ as compared to CO_2_ diminishes the effect of atmospheric oxygen on the sensor performance.

In addition to the above ionic liquid-based electrochemical CO_2_ sensors, few reports are found on the use of nanomaterial combined with ionic liquid for the detection of CO_2_ gas [66,67,68,69]. Shimoyama *et al.* proposed a gas sensor using a combination of carbon nanotubes (CNTs) and ionic liquid 1-ethyl-3-methyl imidazolium tetrafluoroborate ([EMIM][BF_4_]) [66]. In this study, the authors selectively used ionic liquid [EMIM][BF_4_] and polymer polyethyleneimine (PEI), as both of these materials specially absorb CO_2_ gas. The ionic liquid [EMIM][BF_4_] or the mixture of [EMIM][BF_4_] and PEI dropped on the CNT-FET were applied as a CNT-FET ionic liquid-gate and a surface modification material of CNTs. They proposed that by using the liquid gate, a gate voltage can be applied to the interface between CNTs and the ionic liquid efficiently. It is shown that when ionic liquid [EMIM][BF_4_] on the CNT-FET absorbs CO_2_ gas, CO_2_ reacts with water and PEIs and changes to ions. When a voltage was applied to the ionic liquid, the ions were attracted on to the CNT surface, and thus, CNTs were affected by the change around their surface. The authors proposed that CO_2_ gas can be easily detected by the measurement of the change resulted from the source/drain current flowing through the CNTs. When the ionic liquid absorbs specific gases, the ion types and concentrations change. Applying a voltage to the ionic liquid gate that attracts the ions onto the CNT surface results in the change of the CNT *I-V* property, and this helps in quantifying the CO_2_ concentration. Overall, it is demonstrated that CO_2_ could be effectively detected by the measure of the CNTs resistance change using the mixture of [EMIM][BF_4_] and PEI. The authors proposed that this result was different from that using pure [EMIM][BF_4_]. Use of an ionic liquid-polymer mixture decreased the CNT resistance, because the amino group of PEI transferred charges to the CNTs. Further, the authors reported a CO_2_ gas sensor based on a field-effect transistor (FET) with a graphene channel and ionic liquid gate [67]. Graphene is observed to be supremely sensitive to its surroundings due to its high carrier mobility and surface-to-volume ratio. It is shown that the current-voltage characteristic of the graphene-FET changed in proportion to the logarithm of CO_2_ concentration. The proposed grapheme FET-based sensor selectively detects low concentrations of CO_2_ gas at a low gate voltage. The above device is able to detect 4000 ppm of CO_2_ at a gate voltage below 1 V. Further, the authors have proposed that optimization of the FET design and ionic liquid configuration can improve its sensitivity for CO_2_ detection.

Jin *et al.* have used poly-ionic liquid (PIL)-wrapped single-walled carbon nanotubes for CO_2_ sensing [68]. In the design of the sensor, they synthesized PIL poly[1-(4-vinylbenzyl)-3-methylimidazolium tetrafluoroborate] by a conventional free-radical polymerization method from polymeric ionic liquid monomer. Further, they prepared a homogenous suspension of PIL-wrapped single-walled carbon nanotubes (SWNTs) by grinding SWNT powder in concentrated PIL solution. This PIL-wrapped SWNT electrochemical sensor exhibited superior sensitivity toward CO_2_ with a very low detection limit of 500 ppt. This CO_2_ sensor is found to be reproducible, highly selective and resistant to the interference of relative humidity and offers promising potential for real-time monitoring of CO_2_ with high sensitivity in some special circumstances. Further, Koziej *et al.* proposed a CO_2_ sensor based on tetraalkylammonium-based poly(ionic liquids) (PILs) that are able to absorb particularly large amounts of CO_2_ [69]. The authors used poly[(*p*-vinylbenzyl)-trimethylammonium hexafluorophosphate] (P[VBTMA][PF_6_]) along with inorganic nanoparticles lanthanide-oxycarbonates (La_2_O_2_CO_3_), having an outstanding CO_2_ adsorption capacity. In the design of the sensor, they engineered highly conductive channels localized at the interface between P[VBTMA][PF_6_] and La_2_O_2_CO_3_ nanoparticles. In this approach, the key aspect lies in precise tuning of the conductivity of the composites by taking advantage of the synergistic interaction at the interface between P[VBTMA][PF_6_] and La_2_O_2_CO_3_ nanoparticles. It was observed that the above composites with 70 wt% La_2_O_2_CO_3_ show a decrease in the resistance (implying an increase in conductance, measured from electrical impedance spectroscopy) when exposed to CO_2_ at room temperature and in humid conditions. These composites show a further increase in the conductivity when exposed to pulses of CO_2_ between 150 and 2400 ppm. This work provides a simple strategy to achieve an enhancement of the electrical properties required for the utilization of PIL-based CO_2_ sensors.

## 4. Ionic Liquid-Induced Electrochemiluminescence in CO_2_ Sensing

**Figure 3:**
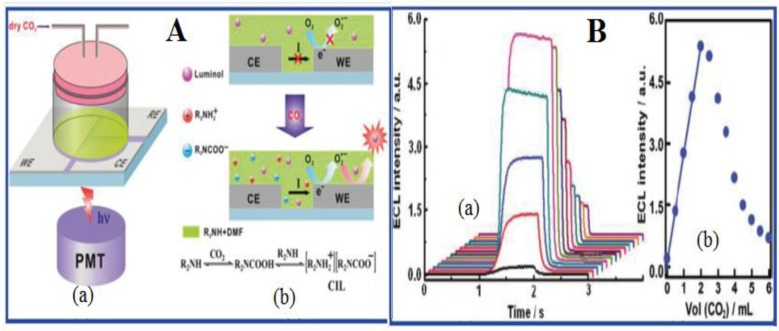
(**A**) (**a**) The schematic diagram of the electrochemiluminescence (ECL) sensor for CO_2_ and (**b**) the principle of the CO_2_ sensor based on ionic liquid-induced ECL; (**B**) (**a**) ECL responses of luminol-O_2_ obtained after injecting various volumes of CO_2_ (100 v/v%) and (**b**) plots of ECL intensity *vs.* the volume of CO_2_ (100 v/v%). ([70] reprinted (adapted) with permission from Chi Y. *et al.* (2011) Anal. Chem. 83: 6862–6867. Copyright (2011) American Chemical Society). CIL, carbamate ionic liquid.

In addition to the above ionic liquid-based CO_2_ sensors where the detection scheme is based solely on either optical (mostly luminescence based) or electrochemical methods, a report is found on electrochemiluminescence-based detection of CO_2_, where a combination of both optical and electrochemical methods is used [70]. Chi *et al.* proposed a CO_2_ sensor based on ionic liquid-induced electrochemiluminescence (ECL) [70]. ECL is a combined process of electrochemistry and chemiluminescence in which species generated at the electrode surface undergo electron-transfer reactions to form excited states that emit light [82,83]. ECL sensors are shown to have the advantages of both electrochemical- and chemiluminescence-based sensors, such as high sensitivity, simple instrumentation and easy control [82,83]. In this ECL-based CO_2_ sensor, the authors have shown that the electrochemiluminescence of the luminol-O_2_ system in an electrolyte-free solution of *N*,*N*-dimethylformamide (DMF) and dipropylamine (DPA) is induced by the formation of a carbamate ionic liquid (CIL) from the reaction between CO_2_ and DPA, which forms the basis of ECL-based CO_2_ detection (Figure 3A).

In this sensor design, luminol acts as the ECL luminophore, and the maximum ECL response was found at a 1:1 volume ratio of DPA to DMF that is used for solubilizing luminol. It is important to mention that no current and ECL emission were observed from such a sensing solution containing luminol, DPA and DMF in the absence of CO_2_, since no conductive ion exists in the solution. However, the injection of CO_2_ gas into the above system leads to the reaction between CO_2_ and DPA to form a conductive CIL that induces strong ECL reactions of the luminol-O_2_ system. It was shown that when dry and pure CO_2_ were injected into the luminol-O_2_ ECL system at a fixed flow rate, the ECL response of the luminol-O_2_ system is linearly dependent on the CO_2_ concentration before getting saturated after reaching the optimum CO_2_ concentration. The ECL intensity was observed to increase with increasing the amount of CO_2_ in the range of 0–2.0 mL and then reaches a maximum at 2.0 mL followed by a decrease with further injection of CO_2_ gas into the sensor (Figure 3B). It is noteworthy that the above ECL sensing method shows several advantages in the detection of CO_2_, such as high selectivity, high safety, a wide linear response range and good sensitivity. This ECL sensor can sense CO_2_ in a short time, allows quantification of CO_2_ over a wide concentration range and is free of interferences from other gases. This CO_2_ sensor was found to have a detection limit of 80 ppm. This is the first reported ionic liquid-induced ECL sensor for CO_2_ gas detection.

## 5. Conclusions

A rapidly-emerging field in advanced sensor research involves the development of sensors and diagnostic devices centered on ionic liquids as an alternative to molecular solvents and conventional materials for the detection and quantification of CO_2_. A cautionary note on the hygroscopic nature of the most ionic liquids must always be kept in mind. A thoroughly dried ionic liquid is desired in CO_2_ sensing applications to minimize water-CO_2_ interactions. Further, the thermal stability of ionic liquids for CO_2_ applications must be considered critically, as many ionic liquids show a significantly lower thermal stability than inorganic materials. It is also important to mention in this context that temperature also affects the viscosity of the medium, which, in turn, may destabilize the sensor. The fact that an ionic liquid may be synthesized for a specific application by simply manipulating its key physicochemical properties as a result of the appropriate selection of cation and anion combinations should also be considered carefully. The manipulation of the physicochemical properties of the ionic liquids is not simple, as in several instances, the cation/anion combinations do not afford desired outcomes. Finally, it is important to mention that the use of ionic liquids in the sensing of biomolecules is in its early stages. However, the rapid growth in this field is fairly evident from the current literature. This review highlights the use of ionic liquids as both optical and electrochemical CO_2_ sensors. Most of the optical-based CO_2_ sensors are shown to have used fluorescence emission as the important tool, and some reports are found to use UV-VIS absorbance and surface plasmon resonance (SPR) for CO_2_ detection. Most of the electrochemical CO_2_ sensors are based on cyclic voltammetry (showing a change in *I-V* response), amperometry (based on current change) and electrical impedance (based on the change in resistance) measurements. It is important to highlight that not all of the CO_2_ sensors are purely ionic liquid based; some of them utilize other materials, along with ionic liquids, such as polymers, nanomaterials and several other appropriate compounds, for the detection of CO_2_. The sensing mechanisms of the ionic liquid-based CO_2_ sensors are described in detail in this review. The principle, design, sensitivity and detection limits are also highlighted. This review is supposed to be helpful to researchers working in the area of ionic liquids, as well as CO_2_ detection.
